# Simultaneous oscillatory encoding of “hot” and “cold” information during social interactions in the monkey medial prefrontal cortex

**DOI:** 10.1016/j.isci.2024.109559

**Published:** 2024-03-25

**Authors:** Fabio Di Bello, Rossella Falcone, Aldo Genovesio

**Affiliations:** 1 Department of Physiology and Pharmacology, Sapienza University of Rome, Rome, Italy; 2 Leo M. Davidoff Department of Neurological Surgery, Albert Einstein College of Medicine Montefiore Medical Center Bronx, Bronx, NY, USA

**Keywords:** Neuroscience, Social sciences

## Abstract

Social interactions in primates require social cognition abilities such as anticipating the partner’s future choices as well as pure cognitive skills involving processing task-relevant information. The medial prefrontal cortex (mPFC) has been implicated in these cognitive processes. Here, we investigated the neural oscillations underlying the complex social behaviors involving the interplay of social roles (Actor vs. Observer) and interaction types (whether working with a “Good” or “Bad” partner). We found opposite power modulations of the beta and gamma bands by social roles, indicating dedicated processing for task-related information. Concurrently, the interaction type was conveyed by lower frequencies, which are commonly associated with neural circuits linked to performance and reward monitoring. Thus, the mPFC exhibits parallel coding of both “cold” processes (purely cognitive) and “hot” processes (reward and social-related). This allocation of neural resources gives the mPFC a key neural node, flexibly integrating multiple sources of information during social interactions.

## Highlights

• Beta and gamma bands account for social roles independent of interaction type
• Low frequencies (below 20 Hz) depend on the interaction type
• The mPFC allows simultaneous and parallel coding of “cold” and “hot” processes

## Graphical abstract

**Figure:**
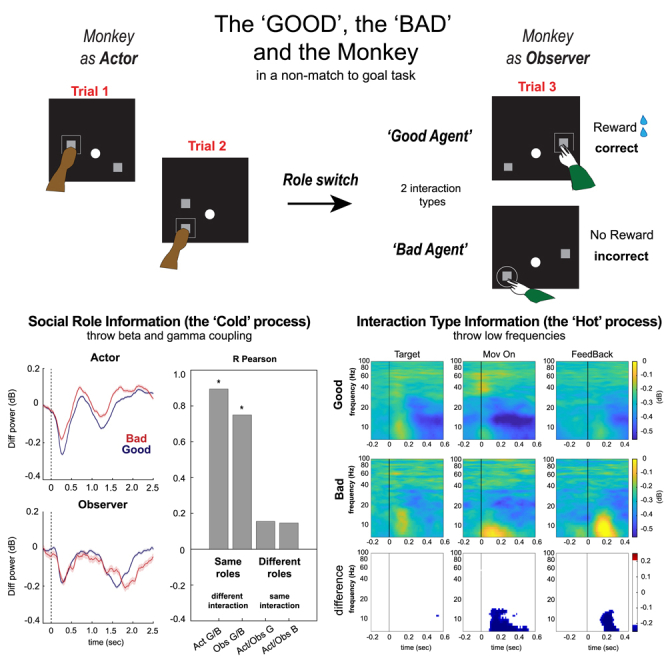

## Introduction

In primates, including monkeys, social interaction seems to be a very present feature in their life, consisting of moments of cooperation and competition. Rhesus monkeys are capable of interfacing with social challenges thanks to their ability to use information gained from monitoring the outcomes of others’ actions and choices^1^^,^^2^ and to regulate their behavior during both social interactions^3^^,^^4^ and social learning.^5^^,^^6^^,^^7^^,^^8^^,^^9^ They can also discern others’ perceptions based on their gaze,^10^ make pro-social decisions,^11^ and detect false beliefs.^12^ Thus, social abilities span a spectrum that encompasses purely cognitive information processing (“cold” cognitive functions) and the processing of reward- and emotion-related information (“hot” cognitive functions; see^13^ for a detailed review).

In recent neurophysiological studies, including our own, it has been shown that the medial prefrontal cortex (mPFC) has neurons that play a role in social behavior,^7^^,^^14^^,^^15^^,^^16^^,^^17^ monitoring reward outcomes and values,^7^^,^^17^^,^^18^^,^^19^ and in representations of self and other.^20^ This brain area is also associated with more analytical cognitive functions including decision-making,^21^^,^^22^ conflict monitoring,^23^ working memory (WM),^24^^,^^25^^,^^26^ attention,^27^^,^^28^ and in the selection of response tactics.^29^ As such, the mPFC might serve as a crucial neural node that integrates and encodes multiple sources of information during social interactions.

To examine the interplay of the different neural dynamics underlying social behavior, we analyzed the local field potentials (LFPs) recorded from the mPFC of two monkeys performing a nonmatch-to-goal task. In this task, monkeys switched the Actor and the Observer social role with a human agent in different trials.^14^^,^^15^ Critically, the monkeys could be engaged in two distinct types of interaction depending on the human partner they were interacting with: the ‘Good Agent’ who always made correct choices, and the ‘Bad Agent’ who always chose incorrectly. This allowed for the investigation of the monkeys’ interactions in different relational contexts with different human partners that could either align with or conflict with the monkeys’ own goals and intentions. This task paradigm also encouraged active involvement in social interaction as the monkeys had to closely monitor their partner’s choices during the observation phase to successfully transition into the role of the actor when roles were switched. LFP signal comprises several band-limited components that are associated with various functions and processing pathways.^30^^,^^31^^,^^32^ Through the analysis of this signal, we obtained important insights into the complex dynamics of social interactions.

First, we identified a characteristic modulation of the beta (20–30 Hz) and gamma (45–100 Hz) bands during specific task events related to the role played by the monkeys either as actor or observer, in different types of interaction. Our results are consistent with the notion of a different task-relevant information processing, specific to each social role. In the Actor role, the implementation of the non-match to goal rule is essential for target selection, but less so in the part of the trial that mainly involves the execution of actions based on the selected target. Conversely, in the observer role, the acquisition and retention of information about the human choices is critical for response selection when the role changes in subsequent trials. We propose that a dedicated use of working memory (WM) for social roles may play a role in the manifestation of this frequency coupling. Second, we observed that lower frequencies (below 20 Hz), which are typically associated with neural networks involved in performance monitoring, were informative of the type of interaction. Thus, we observed a parallel coding of two separate neural pathways: one associated with “hot” processes (that is, reward and social-related information processing) and the other with “cold” cognitive processes (that is, task-related information processing). This modular allocation of neural resources might confer a computational advantage for the efficient integration of social information to assist simultaneous access and use of different types of information.^33^ At the same time, maintaining separate neural circuits helps minimize potential interference between ongoing cognitive processes. Overall, our results provide evidence for the role of mPFC as a neural hub that flexibly and simultaneously encodes and integrates multiple sources of information during social interactions.

## Results

### Behavior

**Figure 1:**
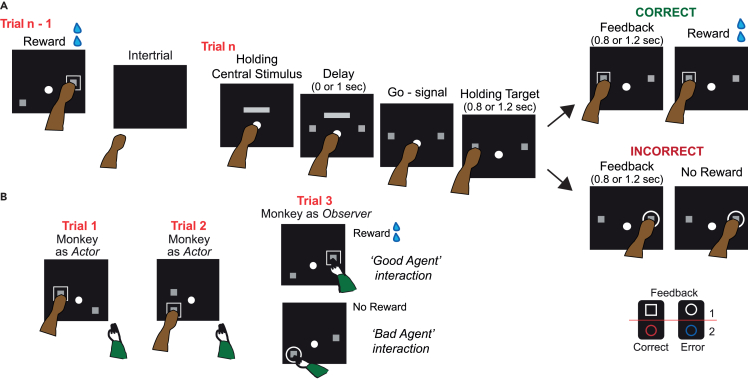
Task events and interaction with human agents (A) Timeline of events in a single trial of the NMTG task. The monkey (the Actor in this example) holds a central stimulus (white circle) until the gray horizontal bar at the top disappears, which serves as a Go-signal and triggers a reaching movement toward one of the two spatial targets (gray squares, on the center left and center right in this example). The chosen target is held until feedback is displayed indicating whether the choice is correct or incorrect. If the chosen target is correct, the monkey receives a liquid reward. The black rectangles represent the video screen. On the right side of the image, the four feedback stimuli used in pairs indicate the correctness or incorrectness of the choice. (B) The monkey interacts with a human agent sitting to its right, alternating between the roles of Actor and Observer. In this example, the monkey as Actor performs two consecutive trials, 1 and 2 (left panels). Trial 3 is performed by the human agent and the monkey is the Observer. The human agent can be a Good Agent (top panel in B) or a Bad Agent (bottom panel in B). The Good Agent performs the trial correctly, and the monkey receives a liquid reward. The Bad Agent chooses the incorrect target. The green arm represents the human arm.

Two monkeys performed the nonmatch-to-goal task while they were interacting with a human partner. Monkey and humans alternated as Actor and Observer (Figure 1A; see STAR Methods for detailed description). Human intervention was not cued. The human partner unpredictably decided when to start a trial by reaching for the screen with his arm only during the intertrial period, ready to touch the central stimulus on the screen after it was turned on.

The monkey could face two different types of interaction, based on whether the ‘Good Agent’ or the ‘Bad Agent’ was at its side. The Good Agent performed the task correctly, allowing the monkey to receive the reward regardless of which of the two (‘Good’) human partners was involved (Figure 1B, middle panel). The Bad Agent always performed incorrectly, thus preventing the monkey from receiving the reward (Figure 1B, right panel). In both types of interaction, whether with the Good Agent or the Bad Agent, the monkey switched between social roles: as Actor, when it was the one performing the trial, and as Observer when the human was performing the trial (Figure 1B).

**Figure 2:**
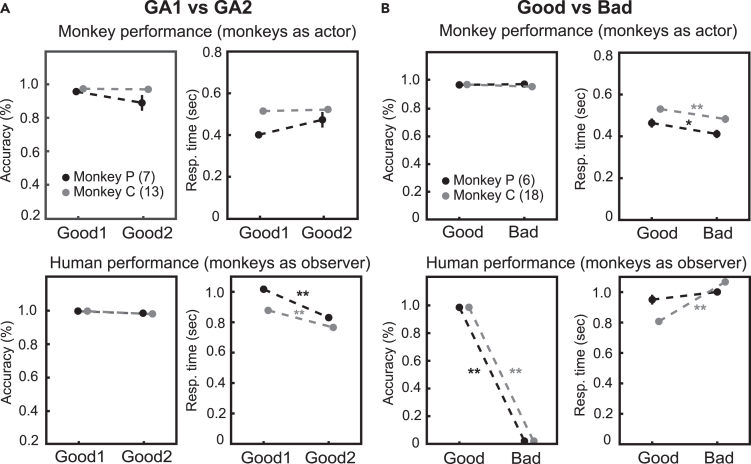
Behavioral Performance in different types of interaction (A) Accuracy and Response time (reaction-movement time) of monkeys (top left and right panels, respectively) and humans (bottom left and right panels, respectively) during the interaction between the monkey and the two Good Agents, GA1 and GA2. Data from sessions in which GA1 and GA2 alternate within the same session. (B) Accuracy and Response time (reaction-movement time) of monkeys (top left and right panels, respectively) and humans (bottom left and right panels) during the interaction between the monkey and at least one Good Agent and the Bad Agent. Data from sessions in which the Good and the Bad Agents alternate within the same session. Black and gray dots indicate the behavioral values of monkey P and monkey C, respectively. The number within the parentheses indicates the number of sessions included in the analyses. Statistical analysis was performed using the Kruskal Wallis nonparametric test, with significance indicated as ∗p < 0.05 and ∗∗p < 0.001. Error bars represent mean ± SD.

In total, three human subjects participated in the experiment as human agents, always maintaining the same role. The Good Agent role was performed by two subjects, GA1 and GA2, respectively. The Bad Agent role was performed by one subject, BA. Each subject, one at a time, interacted with the monkey. The agents alternated unpredictably. The Good Agents could perform 1, 2, or 3 consecutive trials, and rarely 4, while the Bad Agent always performed 1 trial (see Data S1 for a description of the results). It was not always the case that all three human agents participated in the same session. In some cases, there were only two agents, either both good or one good and one bad, or only one agent (either one GA or BA). To test whether the monkeys’ performance was affected by the specific human subject performing the task, given that the two agents had the same social role of Good Agent, we used a subsample of sessions in which both GA1 and GA2 interacted with the monkeys (Figure 2A; see Table 1). The performance of the two monkeys (Figure 2A – top panels) was not affected by which human agent was sitting next to them, both in terms of accuracy (mean ± std, monkey P with GA1 0.96 ± 0.09 vs. with GA2 0.89 ± 0.47, 7 sessions, Kruskal-Wallis nonparametric test, p = 0.44; monkey C with GA1 0.97 ± 0.6. vs. with GA2 0.97 ± 0.1, 13 sessions, Kruskal-Wallis nonparametric test, p = 0.54) and response times (mean ± std, monkey P with GA1 401.9 ms ± 16.2 vs. GA2 473.0 ms ± 37.2, 7 sessions, Kruskal-Wallis nonparametric test, p = 0.14; monkey C with GA1 511.8 ms ± 13.5 vs. with GA2 518.8 ms ± 15.7, 13 sessions, Kruskal-Wallis nonparametric test, p = 0.59; Figure 2A - top panels). Moreover, as expected, we found that both Good Agents were very accurate when performing the task with each monkey (mean ± std, with monkey P: GA1 1.0 vs. GA2 0.99 ± 0.9, 7 sessions; with monkey C: GA1 1.0 vs. GA2 0.98 ± 0.7, 13 sessions; Figure 2A - bottom panels). Both Good Agents were faster with monkey C than with monkey P (mean ± std, GA1 with monkey P 1002 ms ± 11.7 vs. with monkey C 878 ms ± 20.0, p < 0.01; GA2 with monkey P 830 ms ± 20.7 vs. with monkey C 766 ms ± 10.0, 13 sessions, Kruskal-Wallis nonparametric test, p < 0.05; Figure 2A - bottom panels). However, GA2 was faster than GA1, regardless of the monkey beside (mean ± std, with monkey P, GA1: 1002 ms ± 11.7 vs. GA2: 830 ms ± 20.7, 7 sessions, p < 0.01; with monkey C, GA1: 878 ms vs. GA2: 766 ms ± 10.0, 13 sessions, Kruskal-Wallis nonparametric test, p < 0.001; Figure 2A, bottom panels).

**Table 1:** Number of sessions for each type of analysis performed

TYPE OF ANALYSIS	MONKEY P	MONKEY C
Comparison between GA1 and GA2	7/23 sessions Visually Selective Channels: 21	13/32 sessions Visually Selective Channels: 27
Comparison between BAD and GOOD	6/23 sessions (1 with GA1, 2 with GA2, 3 both GA1 e GA2) Visually Selective Channels: 16	18/32 sessions (3 with GA1, 8 with GA2, 7 with both GA1 and GA2) Visually Selective Channels: 44

To test whether the monkeys’ performance was affected by the type of interaction, that is, whether the monkey interacted with humans with a different role of Good or Bad, we analyzed sessions in which the monkey worked with both the Bad Agent and at least one Good Agent (Figure 2B; see Table 1). The accuracy of the two monkeys was not affected by the role of the interacting human agent (mean ± std, monkey P with GA 96.5 ± 0.6 vs. BA 98.3 ± 0.8, 6 sessions, Kruskal-Wallis nonparametric test, p = 0.11; monkey C with GA 97.2 ± 0.5 versus BA 94.9 ± 1.9, 18 sessions, Kruskal-Wallis nonparametric test, p = 0.26; Figure 2B – top panels). In contrast, monkeys were significantly faster when interacting with the Bad Agent (mean ± std, monkey P with GA 463.6 ms ± 21.2 vs. with BA 411.3 ± 14.0, 6 sessions, Kruskal-Wallis nonparametric test, p < 0.05; monkey C with GA 535.0 ms ± 13.5 vs. with BA 487.3 ms ± 19.1, 18 sessions, Kruskal-Wallis nonparametric test, p < 0.01).

By task design, the Good Agent was very accurate with each monkey (mean ± std, with monkey P: 0.99 ± 0.4, 6 sessions; with monkey C: 0.98 ± 0.7, 18 sessions; Figure 2B, bottom panels). The Bad Agent always made incorrect choices with both monkeys. The Good Agent showed significantly faster responses than the Bad Agent with monkey C (mean ± std, GA = 816.8 ms ± 11.9 ms vs. BA = 1104.4 ms ± 22.1 ms, 18 sessions, Kruskal-Wallis nonparametric test, p < 0.001), but was only marginally faster with monkey P (mean ± std, GA = 950.5 ms ± 32.0 ms vs. BA = 1034.2 ms ± 26.5 ms, 6 sessions, Kruskal-Wallis nonparametric test, p = 0.15). Both monkeys performed well after the trial performed by the human agents, demonstrating the monkey’s ability to monitor the human partner’s choice in both types of interactions (see Data S2 for a description of the results).

To assess whether the interactions with the Bad Agent were underrepresented in the data compared to those with the Good Agent, we compared the number of interactions carried out by both social roles in the sessions that contained both types of interactions. The average number of turns (that is, the trials made by the human agent after a trial made by the monkey) for the Good Agent was on average n = 24.8 ± 12.1 and n = 28.2 ± 10.6 for monkeys P and C, respectively. The average number of turns for the Bad Agent was n = 21.5 ± 2.3 and n = 16.7 ± 8.1 for monkeys P and C, respectively (when both the two Good Agents participated in a recording session, we summed their turns considering as if it were the number of turns of a single Good Agent). We believe that the data analyzed come from sessions in which the turns involving the Bad Agent were not so rare that the monkeys considered the Bad Agent trials to be outliers.

### Neurophysiology

**Figure 3:**
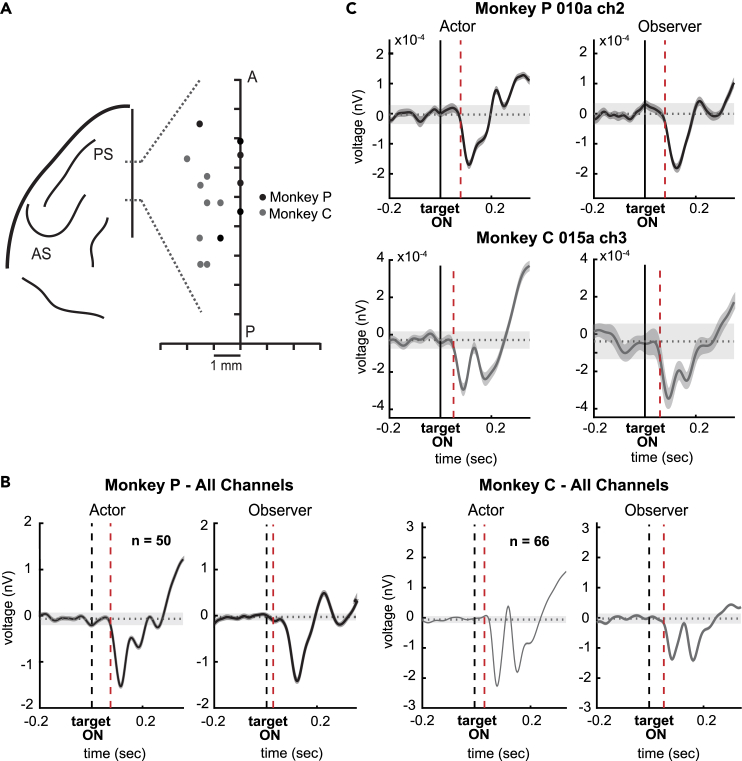
Recording sites and channels voltage modulation (A) Top view of the left frontal lobe derived from the MRI of monkey 2. Two gray dashed lines represent the estimated boundaries of the mPFC area in the anteroposterior axis of the brain. PS, principal sulcus; AS, arcuate sulcus. Distribution of the recording sites whithin the area for monkey P (dark gray dots) and monkey C (gray dots). (B) Average voltage modulation considering all recorded channels from 0.2 s before and 0.35 s after target presentation in Monkey P (top panels) and Monkey C (bottom panels), when the monkey is the Actor (left panels) and the Observer (right panels). The dashed red line indicates the time when the voltage exceeds the ± 2SD threshold (shaded gray band), with the SD calculated within the time window [-0.2 0] after target presentation. (C) Voltage modulation of two example channels. Same conventions as in B.

In our previous work, during the Nonmatch-to-goal task, mPFC single neurons showed a robust activation both when monkeys performed the task and observed a human agent performing the task.^14^^,^^15^ Here, for all the artifact-free channels, we checked whether the voltage at targets presentation differed between the two conditions in which the monkey was the Actor and the Observer. Each channel was considered visually selective if its voltage was modulated by > 2SD from baseline for 50 ms immediately after the targets were presented (see STAR Methods; Figure 3B).

Both monkeys had a high proportion of visually selective channels in the Actor (monkey P: 47/50 (0.94); monkey C: 65/66 (0.98)) and in the Observer role (monkey P: 48/50 (0.96); monkey C: 65/66 (0.98)). The number of visually selective channels recorded when the monkey was in the two different social roles did not differ for each monkey, respectively (monkey P: Actor = 0.94 vs. Observer = 0.96, n channels = 50; Kruskal–Wallis nonparametric test, p = 0.67; monkey C: Actor = 0.98 vs. Observer = 0.98, n channels = 66; Kruskal–Wallis nonparametric test, p = 1). This result suggests that the monkeys were similarly engaged in monitoring the task execution in the two social roles. Only one channel did not show significant modulation during either the actor or the observer role (in Monkey P), and it has been excluded from subsequent analyses.

#### The LFP signal accounts for the agent type and not for the agent identity

We wondered whether the LFP dynamics in the mPFC encoded the role of Good Agent per se, with two agents sharing the same role, or whether there was also a differentiation between the two agents based on their identity. To this aim, we analyzed the LFP spectrograms of the two monkeys as they interacted with each Good Agent as both Actor and Observer. For this purpose, we used the sessions in which the monkey performed the task with both Good Agents. In both the actor and the observer role, the permutation test performed on each time-frequency bin showed no main differences when the monkey interacted with either GA1 or GA2 (see STAR Methods; see Figure S1). The LFP signal of mPFC was not affected by the identity of the human agent with the same Good Agent role. Since no significant differences were found between GA1 and GA2, both trial types were included in the Good Agent spectral analyses.

#### LFP dynamics accounts for the social role: Actor versus Observer

To study the neural dynamics underlying the monkey’s role as Actor and Observer, we analyzed the evolution of alpha (7-15Hz), beta (20–30 Hz), and gamma (45–100 Hz) frequency bands in a 3 s time window, from 0.5 s before the presentation of the targets to 2.5 s after the target onset, which included the completion of the reaching movement toward the chosen target.

We examined the LFP dynamics when the monkey was the Actor and the Observer during the interactions with both the Good Agent and the Bad Agent. Our analysis focused on the dynamics of alpha oscillations and the coupling of beta and gamma bands, respectively. Our choice to investigate the beta/gamma coupling is motivated by its well-established association cognitive control in PFC.^34^^,^^35^^,^^36^ Specifically, we investigated the alpha dynamic and the interrelationship between beta and gamma responses that emerged after the target presentation and around the reaching movement.

#### Alpha band

##### Good agents interactions

This analysis includes all sessions in which the monkey interacted with at least one Good Agent. Following the target presentation, we found a rapid increase in alpha relative power in both the Actor and Observer roles (Figure S2). Subsequently, there was a gradual decrease in alpha power in both monkeys. Interestingly, we observed opposite modulations of alpha in response to movement onset depending on the social role. In the Actor role, we observed a decrease in alpha activity following the movement onset (in both monkeys, Kruskal-Wallis nonparametric test, p < 0.001). Conversely, in the Observer role, we observed an increase in alpha power following the movement onset (Monkey P: p < 0.001; Monkey C: p < 0.03, Kruskal-Wallis nonparametric test).

##### Bad agent interactions

This analysis included all sessions in which the monkey interacted with the Bad Agent. Following target presentation, we observed a rapid increase in alpha relative power in both the Actor and Observer roles (Figure S2), followed by a tonic decrease in both monkeys. We also observed different responses of alpha modulation depending on the social role and in response to the onset of movement. In the Actor role, a significant decrease in alpha activity was observed in Monkey P (Kruskal-Wallis nonparametric test, p < 0.001), while Monkey C exhibited a weak decreasing trend (Kruskal-Wallis nonparametric test, p = 0.18). Conversely, in the Observer role, an increase in alpha power was noted in Monkey P (Kruskal-Wallis nonparametric test, p < 0.001), and a similar trend was observed in Monkey C (Kruskal-Wallis nonparametric test, p < 0.08).

In summary, we found a decrease in alpha power in response to the movement onset in the Actor role, whereas an increase in alpha power followed movement onset in the Observer role, although more pronounced in monkey P. These patterns remained consistent for interactions with both Good Agents and Bad Agents, although we found only a weak trend of alpha reduction in the Bad Actor role in Monkey C. In addition, we observed greater alpha activations during the interactions with the Bad Agent compared to those with the Good Agent, during both epochs for Monkey C and after Movement Onset for Monkey P. We will explore this comparison further in the following section, using sessions in which the monkey engaged in both types of interactions.

#### Beta and gamma bands

**Figure 4:**
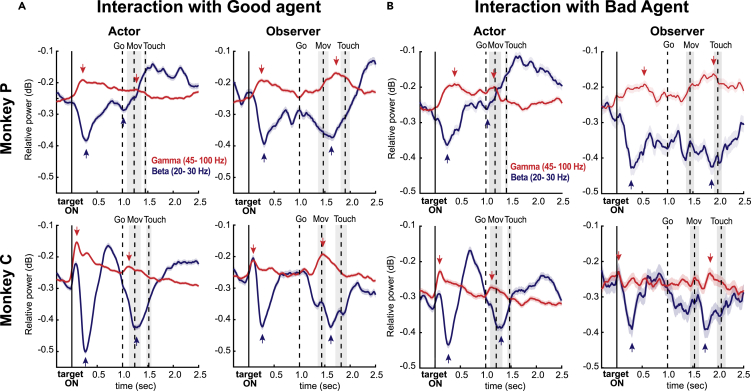
LFP band dynamics of social roles in different types of interaction Average relative spectrograms, normalized to baseline, of beta (blue) and gamma (red) bands when the monkey is the Actor and the Observer during the interaction with the Good Agent (A) and the Bad Agent (B). The vertical line indicates the target onset time (time 0). The vertical dotted lines correspond to the Go-signal, the average Movement onset and the Touch onset time. The gray shaded bars indicate the plus/minus standard error (SE). The vertical blue (red) arrows represent the local minimum (maximum) of beta (gamma) oscillations, calculated within the time window [0, 0.6] and [-RT Touch], respectively.

After target onset, we detected two distinct and anticorrelated modulatory phases of the beta and gamma signals, probably corresponding to distinct computational phases. Each modulatory phase consisted of a gamma synchronization and a beta desynchronization, coupled around two critical epochs of the task (i.e., target onset and movement onset, see Figure 4). The strength of these opposing modulations was associated with social roles, regardless of the specific interaction in which the monkey was involved. In the Actor role, there was increased activation of both beta and gamma resources in the early phase of the trial (after the target onset) compared to the late phase of the trial (around movement onset). In contrast, in the Observer role we observed a greater gamma engagement in the late part of the trial compared to the early part, while the beta deflections did not differ between the two critical epochs of the task (see Data S3 for a description of the results).

When comparing the latencies around movement onset between the Actor and Observer in both interaction types, although the order of neural response offsets differs between the two monkeys (i.e., the beta peak precedes the gamma peak in Monkey P, while the opposite is observed in Monkey C), we found that both beta gamma responses peaked earlier in the Actor role compared to the Observer role in both monkeys (Kruskal-Wallis nonparametric test, p < 0.001 for both monkeys in both interaction types). Overall, the event-locked fluctuations that occurred around the reaching movement were delayed in the observer role compared to the actor role.

##### Beta-gamma response patterns

Although the statistical significance of the differences between beta and gamma peaks in different conditions suggests a characteristic involvement of these frequency bands for different social roles, regardless of the interaction type, it is possible that the overall response pattern (the difference between gamma and beta) does not go in the same direction. To explore this further, we examined whether the differences in overall beta-gamma response patterns were more similar when comparing the same social roles in different interactions or when comparing different social roles within the same social interaction. We found that the beta-gamma response patterns were more correlated between the same social roles than between different social roles (Monkey P, Actor Good/Bad r = 0.84, Observer Good/Bad r = 0.71, Actor/Obs in Good r = 0.40, Actor/Obs in Bad r = 0.32; Monkey C, Actor Good/Bad r = 0.84, Observer Good/Bad r = 0.71, Actor/Obs in Good r = 0.40, Actor/Obs in Bad r = 0.32), as shown in Figure S4. This finding supports the notion that the beta-gamma coupling response pattern is associated with the processing of role information, regardless of the type of interaction.

##### Target information through beta oscillations

Prefrontal beta oscillations have been linked to working memory (WM) engagement.^26^^,^^35^^,^^37^ To investigate the role of beta activity in the retention of information across trials, we used a decoding technique (see STAR Methods). Specifically, we examined whether information regarding the correct target in trial n-1 was maintained at the outset of trial n. Our results indicate that beta activity continued to encode the previous correct target position, thereby providing valuable information for applying the non-match rule when transitioning from the observer to the actor role. Figure S5 shows a significant decoding accuracy for the correct target in trial n-1 (also for the incorrect target in trial n) across the presentation of the targets for both the Good Agent (Figure S5: from -200 ms to 50 ms for Monkey P and from -200 ms to 50 ms for Monkey C, permutation test, p < 0.01) and the Bad Agent (Figure S5: at -200 ms and from -100 ms to 50 ms for Monkey P and from -50 ms to 0 ms for Monkey C, permutation test, p < 0.01) interactions.

#### LFP signature of interaction type: Good Agents versus Bad Agents

We tested whether the LFP signal differentiated between the type of interaction with the Good Agent and the Bad Agent. Moreover, we asked whether this putative difference would emerge when the monkey was the Actor or the Observer. Since the movement onset timing was affected by temporal variability, the analyses were locked to the target presentation and movement onset separately. We examined the neural activity that occurred during the interaction with at least one Good Agent and the Bad Agent, one at a time.

##### Target and movement onset alignments

**Figure 5:**
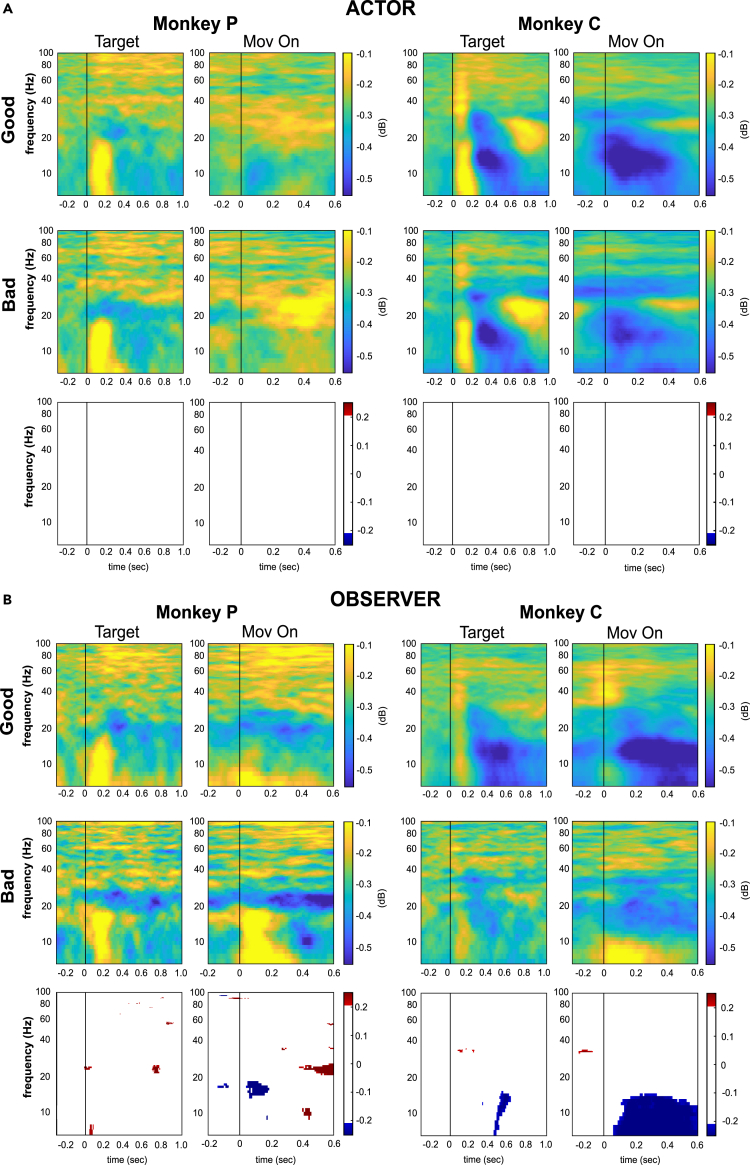
Grand average time-frequency plot of social roles in different types of interaction Average relative spectrograms, normalized to baseline, of the raw LFPs of monkey P (left panels in A-B) and monkey C (right panels in A-B) during the interaction with Good Agent and Bad Agent, when the monkey is the Actor (A) and the Observer (B). Activity is aligned to Target onset (left panels) and Movement onset (right panels). The bottom panels in A-B show the maps of the difference between the spectrograms of Good Agent and Bad Agent. Colored pixels represent statistically positive (red color scale; p < 0.01) or negative (blue color scale; p < 0.01) differences.

Figure 5A shows the time-frequency maps of each monkey as Actor when interacting with the Good Agent (top panels) and the Bad Agent (middle panels). For each monkey, we found that the spectrograms of the two types of interaction did not show differences in the two epochs analyzed (Figure 5A, bottom panels). Thus, when the monkey was the Actor, the mPFC was unaffected by the role of the partner sitting beside the monkey.

When the monkeys assumed the role of Observer (Figure 5B), significant differences emerged in both monkeys. Following the target presentation, the power of low frequencies (7–20 Hz) and of the gamma band rapidly increased while the beta band power declined in both monkeys. A difference between the two types of interaction emerged in lower frequencies (under 20 Hz), which included the alpha range of frequencies (7–14 Hz), showing higher activation for the Bad Agent in monkey C after ∼0.5 s. In contrast, Monkey P exhibited only a slight increase in activation for the Bad Agent before the starting of the movement at the range of frequencies 17–19 Hz.

After the movement onset, low frequencies power rapidly emerged when the Bad Agent moved toward the (wrong) target, in both monkeys. More specifically, between 14 Hz and 19 Hz for monkey P, and between 7 Hz and 15 Hz for monkey C (Figure 5B).

Thus, the low frequencies of the LFP discriminated the type of interaction after the movement began, showing higher power when the behavior of the Bad Agent became explicit. Interestingly, we also observed these differences during the delay period, before the Bad Agent started moving toward the wrong target.

##### Feedback onset alignment

We also wondered whether the LFP correlates were associated with task-related feedback.

**Figure 6:**
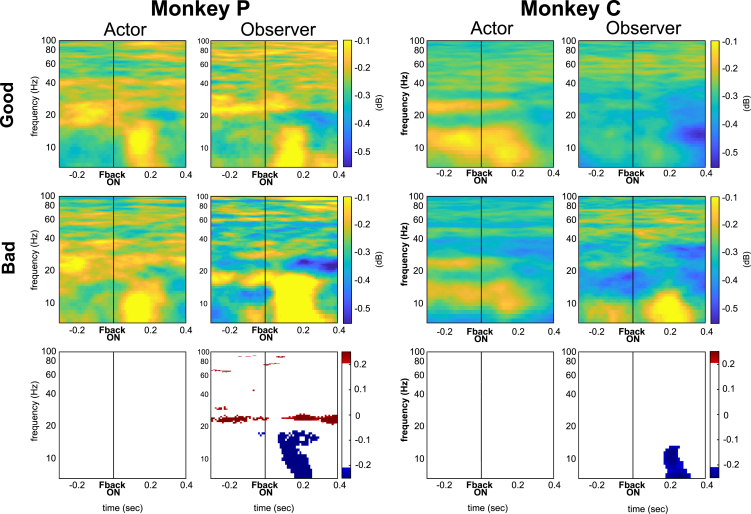
Local field potential signature of error feedback Average relative spectrograms, normalized to baseline, of the raw LFPs of monkey P (left panels) and monkey C (right panels) during the interaction with Good Agent and Bad Agent when the monkey is the Actor and the Observer, aligned to feedback appearance. The bottom panels show the difference between the spectrograms of Good Agent and Bad agents. Colored pixels represent statistically positive (red color scale; p < 0.05) or negative (blue color scale; p < 0.01) differences.

For instance, we compared the two different types of outcome-feedback embedded in different types of interaction. Figure 6 showed an increase in the power of low frequencies in response to feedback in both Good and Bad Agent interactions. No significant differences emerged between these two types of interaction when the monkey was the Actor. In contrast, when the monkey was the Observer, we found significantly greater relative power in favor of the Bad Agent expressed in the alpha frequency ranges in both monkeys (7–19 Hz in monkey P, 7–15 Hz in monkey C). This increased activation emerged after around ∼100 ms in monkey P and ∼200 ms in monkey C from feedback onset. We also noticed increased beta band activation (20–30 Hz) in monkey P before and after the feedback presentation (Figure 6, lower panels).

## Discussion

LFP activity was analyzed to investigate the neural networks underlying the complex social behaviors involved in the interplay between social roles (Actor and Observer) and interaction types (Good Agent’s and Bad Agent’s interaction). To this end, we used a Nonmatch-to-goal task in which the monkey alternated between the actor and observer roles with a human partner who either performed correctly (Good Agent) or incorrectly (Bad Agent) during their turn. The alternation between monkey and human trials was unpredictable, and the number of trials performed by the human agent varied, favoring the continuous engagement of the monkey in observing the partner’s task to successfully transition into the role of Actor when the roles were switched. Both monkeys performed the task very accurately when they were the Actor regardless of the type of interaction. Both monkeys were faster when interacting with the Bad Agent when correction trials were provided. The two Good Agents showed near-perfect performances, with GA2 being faster than GA1. The Bad Agent always performed incorrect trials and was generally slower than the Good Agent.

### Comparable initial visual information between action execution and observation

Performing a task accurately is a reliable indication that the task instructions have been learned and understood. However, assessing learning during observation is more difficult, especially when a subject (i.e., the observer) is not required to explicitly intervene in the actor’s performance. Previous studies have suggested that a human observer may be less engaged in a task than when performing it.^38^^,^^39^^,^^40^ Babiloni et al.,^38^ using electrocorticographic (ECoG) signals, have reported reduced alpha/beta amplitudes in the somatosensory and prefrontal areas during observation compared to execution. In contrast, we did not observe any differences in voltage after target presentation in either the actor or observer role, suggesting a similar level of visual responsiveness between the two social roles. This finding may be attributed to the task’s design, in which each trial depended on the previous one. In other words, each choice made in one trial influenced the choice in the next trial, thus encouraging the monkeys to engage in the task in both social roles. This task feature was not present in the task used by Babiloni et al.,^38^ where each trial did not affect the next trial.

### mPFC does not reflect the partner identity

An important question is whether the mPFC represents social information independently of the partner’s identity based on physical attributes. Although it has been suggested that this area in humans is more involved in evaluating mental states than physical attributes,^41^ it is unclear whether this also applies in the context of social interaction. Our findings revealed that LFP activity in the mPFC did not distinguish between human subjects in the same Good Agent role but rather between the monkey’s social role as an Actor or Observer. In our study, we did not find social agent identity signals as Báez-Mendoza et al. described in the dmPFC at the level of single neuron.^42^ Differences between the two studies may result from the different recording areas, the type of signal acquired, and the difference in the task set-up. Most importantly, only in the task of Báez-Mendoza et al.^42^ the interaction was species-specific. The species-specific nature of the interaction could potentially enhance the monkeys' capacity to differentiate between specific social agents. Importantly, in our task design, the positioning of the human agent relative to the monkey was not directly in front of the monkey, making it difficult for the monkey to discriminate between different human agents based on their physical features. Previous studies have shown that when monkeys are presented with pictures or videos of other monkeys, they spend more time looking at the face and eyes rather than at other parts of the body. Therefore, it is possible that the monkeys never perceived the different agents as different individuals, and from the animal’s perspective the human agent could have been perceived as the same individual appearing multiple times in different roles especially since both agents showed nearly identical performance, in contrast to the findings of Báez-Mendoza et al.^42^ where the subjects showed a different behavior. Whatever the reason for the lack of differentiation of the human identities, we were able to take advantage of it to compare the activity of good and bad agents, which would otherwise have been impossible. The ability of monkeys to recognize facial expressions and differentiate between multiple individuals may be attributed to the presence of neurons that encode facial information.^43^^,^^44^^,^^45^^,^^46^^,^^47^^,^^48^

This initial result allowed us to compare their interactions with both “Good” and “Bad” agents after grouping together the neural signals of the two “Good” subjects. Therefore, we hypothesized that any differences observed within the same social role (Actor or Observer) when interacting with different types of agents (Good or Bad) reflected distinct types of interaction rather than the agent identity.

### Invariant neural coding of social role across interactions

In both social roles (Actor and Observer), beta and gamma bands were anti-correlated over time. These opposite oscillations displayed their main differences in the proximity of two key task events: target presentation and target reaching.

We observed characteristic modulations of the beta and gamma neural networks associated with social role. While the Actor showed the strongest opposite beta-gamma modulations in response to the target’s presentation, the Observer showed a stronger anti-correlated beta-gamma modulation around partner reaching.

Numerous studies have shown that beta oscillations play a critical role in top-down control, acting as an inhibitory filter across the cortex. In the prefrontal cortex (PFC), low beta oscillations are associated with WM engagement, whereas high beta levels are associated with cognitive inhibition (for a review, see^26^). This pattern also holds true for the governing motor control in premotor areas, suggesting a possible functional similarity between cognitive and motor control processes operating at beta frequencies. Other studies have proposed a link between beta-gamma coupling and WM control in the PFC, suggesting a push-pull mechanism in which reduced beta activity facilitates gamma oscillations for sustained encoding of sensory information in WM.^35^^,^^36^^,^^37^^,^^49^ In our study, successful completion of the task required the use of WM in both the Actor and Observer roles, albeit in different epochs of the trial. In the Actor role, monkeys were initially involved in the selection of the correct target through the implementation of the nonmatch-to-position rule, which required them to retrieve and maintain a representation of the correct target position from the previous trial. Thus, the opposite beta/gamma phasic responses observed after the target presentation may indicate temporal integration of choice selection. In contrast, during the target reaching, this pattern of activities was greatly reduced while still present, as the target had already been chosen during the delay period, and planning was being transformed into action. In the Observer role, the information of the human choice has to be monitored for response selection when role switching occurs in subsequent trials. The near-perfect performance of the monkeys after both the Good and Bad agents’ turns confirms the monkeys’ ability to effectively remember the last choice made by the human agent during the observer role. Thus, while the early anti-correlated beta-gamma activity can be considered a neural correlate of the anticipated target choice during the delay period, as shown in our previous studies,^14^^,^^15^ the opposite beta-gamma modulation, which lasts longer during partner reaching, may still reflect memory of the partner choice.^50^ The observation that the beta band encoded information about the target chosen by the human partner during the beginning of the subsequent trial provides further support for this hypothesis. Alternatively, the observed modulations for the human choice could reflect response inhibition during the observation trials. We cannot rule out the possibility that the monkeys inhibited their willingness to respond when observing human behavior, especially during interactions with the Bad Agent. However, contrary to this interpretation, both monkeys in our study never interfered with the human agent’s turn and did not show a tendency to act. Because our task did not involve a non-social computer or ghost control, we cannot verify that the differences in neural activity can also be explained by an inhibitory response to the observation trials.

Overall, the emerging picture suggests a relationship between the relevance of information within each social role and the strength of anti-mirroring beta/gamma, consistent with the idea of a link between the opposite beta/gamma coupling and WM information processing. However, the proposed task elicits the activation of several cognitive processes associated with the PFC that are difficult to isolate and dissociate from each other. For example, the strong beta-gamma coupling following target onset may also be related to decision-making processes^51^ and/or task rule implementation,^52^^,^^53^ the actual target selection,^50^ as well as the task-relevant information processing.^50^ Further research is needed to clarify this aspect.

Interestingly, we observed no differences in beta/gamma neural resource allocation in the interaction with Bad and Good Agents, indicating independence of these circuits from agent type and choice outcome. This finding suggests the existence of a dedicated neural pathway responsible for task-relevant information analysis, operating autonomously from reward and social information.^54^

### Low frequencies account for interaction type

While the interplay between beta and gamma bands specifically accounted for the social roles, lower frequencies also showed different power modulations depending on the type of interaction. Specifically, frequencies below 20 Hz (referred to as low frequencies in the text) showed larger amplitudes when the monkeys observed the Bad agent during different task epochs.

After the reaching movement toward the (incorrect) target, a strong activation in low frequencies emerged, although it manifested in partially overlapping frequencies between the two monkeys. This observation is consistent with previous research on Error-Related Negativity (ERN), which shows a negative deflection observed in event-related potentials following self or other-directed errors in both humans and monkeys.^18^^,^^55^ In the time-frequency domain, the observed motor errors were associated with increased EEG activity in the medial-frontal delta (0.3–4 Hz) and theta (4–7 Hz) frequencies, similar to what has been recorded during self-executed motor errors.^56^^,^^57^^,^^58^^,^^59^^,^^60^ In our study, we showed that intracranial LFP signals in the mPFC contain error signals at higher frequencies, between 7 and 20 Hz in both animals, compared to those observed with EEG signals, resulting from the summation of a large neuronal population.^61^

Increased activity at low frequencies following negative feedback was also observed. This type of activation is consistent with the well-known feedback-related negativity (FRN), an event-related potential evoked by externally signaled errors.^62^^,^^63^^,^^64^^,^^65^ In contrast to the error signal, this signal contained mostly overlapping frequencies across monkeys.

Previous research has proposed a common physiological mechanism for ERN and FRN signals, suggesting a common role in the computation of prediction errors.^66^^,^^67^^,^^68^ These authors proposed a specialization for detecting discrepancies between actual and intended responses or outcomes. Our results are consistent with these hypotheses, suggesting a mechanism capable of detecting unwanted outcomes caused by others and negative feedback that also includes frequencies commonly associated with the alpha range [7–15 Hz]. In light of these considerations, the less well-defined and less overlapping responses to motor errors between the two monkeys may result from the more ambiguous nature of this event in time, which may require different perceptual cues from trial to trial. In contrast, the visual feedback event provides time-locked visual information.

It is noteworthy that a higher expression of low-frequency power was observed even before the partner’s error became explicit, particularly in one monkey during the delay period. In the other monkey, we observed a slight increase in activity in the 17–19 Hz frequency range just before response onset. We believe that this type of coding may not be related to the physical properties of the agent. Instead, it aligns with the idea that this area is involved in monitoring ongoing social interaction by reporting discrepancies between the partner’s actual and expected goals before the partner’s behavior becomes explicit. The lack of overlap between the monkeys’ results may be due to differences in their perception of being involved in a social interaction that could lead to failure or to differences in what and where they look. We cannot test this last hypothesis because the data lack the eye signal in monkey C. This perception does not depend on a visible event but probably depends on the cognitive strategies, subjective perceptions, and interpretations of the individual animals, which, of course, can vary from one monkey to another. As shown by Fascianelli et al.,^69^ in some cases, it is possible to account for individual differences between monkeys in the strategy used in terms of different neural correlates, but this remains the exception. Unfortunately, these factors, although discussed, are beyond our control in our experiment.

### Conclusions

In this study, social interactions were systematically structured around the anticipation of receiving a reward. The Good Agent consistently solved trials correctly, resulting in a reward at the end of each trial. Conversely, the Bad Agent consistently made incorrect choices, resulting in trials without a reward. This experimental design allowed us to link social and motivational aspects to the interaction type variable.^54^^,^^70^

Conventionally, “hot” cognitive processes include emotional responses, social judgments, and decisions influenced by motivations or rewards.^13^^,^^71^^,^^72^^,^^73^^,^^74^ In our study, “hot” networks are those that are influenced by the type of social interaction. We observed a clear modulation of low frequencies (<20 Hz) by social interaction. The presence of signal differences between the Good Agent and the Bad Agent in this range of low-frequency range, even before a clear event associated with future reward (i.e., in the Observer condition, before the partner’s response), suggests that this network also provides information about the quality of the social relationship.

Conversely, “cold” executive functions are generally associated with emotion and reward-free analytical processing of environmental information in the service of goal-directed behaviors. Accordingly, in our study, we refer to processes as “cold” when they deal with task-related information independent of social interaction. In this sense, we found that the beta/gamma networks are social role specific, independent of the interaction type variable.

Recent studies have proposed a distinction between hot (i.e., reward or affective-related) and cold (i.e., purely cognitive) neural networks within the PFC. The lateral PFC and anterior cingulate cortex (ACC) are predominantly associated with cold executive functions (purely cognitive) and can be considered the cold stream. The medial and orbital PFC (VMPFC and OFC) and the ventral ACC constitute the hot stream and are predominantly associated with hot executive functions (emotion, reward, and social cognition). However, recent evidence suggests that this distinction is not rigid, as these networks show extensive interconnectivity and can even show co-activations depending on task characteristics.^75^^,^^76^ We believe that in complex social interaction tasks, such as the proposed one, the simultaneous and accurate encoding of both types of information requires a precise organization of these networks within the mPFC. In this work, we demonstrate parallel encoding of both types of information, while maintaining distinct neural networks using different pathways.

The parallel and simultaneous encoding of cold and hot information may provide the mPFC with a computational advantage for efficient integration of social information. In addition, maintaining separate neural circuits helps to minimize potential interference between ongoing cognitive processes.

### Limitations of the study

Our results showed that mPFC activity does not discriminate between the ‘Good’ human partners, but between the monkey’s social roles as Actor or Observer. This result may be confounded by the experimental design, which does not allow visual contact with the human agent, thus hindering recognition. Another factor preventing such a distinction may depend on the interaction with another species.

We identified a characteristic modulation of the beta (20–30 Hz) and gamma (45–100 Hz) bands associated with the roles of Actor and Observer, independent of the interaction type. We propose that a dedicated use of working memory (WM) for social roles may play a key role in the manifestation of this frequency coupling. However, the simultaneous activation of multiple cognitive processes during the task, often associated with PFC functions, poses a challenge in isolating the specific contribution of these bands to WM operations.

## STAR★Methods

### Key resources table

**Table:** 

REAGENT or RESOURCE	SOURCE	IDENTIFIER
**Software and algorithms**		

Matlab, R2020a	MathWorks	https://it.mathworks.com
Chronux, version 2.00	Chronux Analysis Software	http://chronux.org/

### Resource availability

#### Lead contact

Further information and requests for resources should be directed to and will be fulfilled by the corresponding author, Aldo Genovesio (e-mail: aldo.genovesio@uniroma1.it).

#### Materials availability

No new unique reagents were generated in this study.

#### Data and code availability

• All data available upon request from the lead contact.
• Code, including analysis software, is available from the lead contact upon request.
• Any additional information needed to reanalyze the data reported in this paper is available from the lead contact upon request.

### Experimental model and study participant details

#### Animals

Two male rhesus monkeys (Macaca mulatta), monkey P (10 years old, 9 kg), and monkey C (7 years old, 8 kg) performed in this study. Animal care, housing, and experimental practices complied with Italian (DD.LL. 116/92 and 26/14) and European (Directive 210/63/EU) on the use of non-human primates in scientific research. The research protocol (Central Direction for the Veterinary Service) was approved by the Italian Health Ministry. The housing setup and experimental protocols complied with European regulations on the treatment and utilization of laboratory animals.

#### Surgical techniques

During the training period, a head-holding device was implanted under aseptic surgical conditions. The animals were anesthetized with isoflurane (Abbott Laboratories) through a constant flux of isoflurane/O2 mixture (1–3%, to effect). Antibiotics and analgesics were administered postoperatively. Before the recording started, again under general anesthesia, a recording cylinder (18 mm in diameter) was implanted stereotaxically. Recording sites were localized relative to the principal sulcus and the arcuate sulcus after opening the dura matter for a different experimental protocol involving the same animal in monkey C and based on stereotaxic coordinates in monkey P. Anatomical boundaries are illustrated on a schematic representation of the brain of monkey C in Figure 3A (Falcone et al., 2022).

#### Task

The monkeys were seated in a primate chair 20 cm away from a 19-inch, 800 x 600-pixel video touch screen, with their heads fixed. Monkeys performed the Nonmatch-to-Goal (NMTG) task (Figure 1A). The trial began when the central stimulus (white 7° circle), appeared in the center of the screen. The monkey had to touch the central stimulus within 2 s, otherwise, the trial was aborted, and a new trial started. Upon touching the central stimulus (with the left hand for monkey P and the right hand for monkey C), a horizontal grey bar (18 ° × 10 °) appeared 14° above center. Continuing to touch the central stimulus for 1 s or 1.5 s, two identical targets (filled grey rectangles, 7.1 ° × 7.7 °) appeared in two of four possible screen positions: center left (23.5° left of center), bottom left (17.5° below and 23.5° left of center), center right (23.5° right of center) and bottom right (17.5° below and 23.5° right of center). After the delay period of 0 s (in 11% of trials) or 1 s from the start of the targets, the horizontal bar disappeared triggering the go-signal. Although underrepresented, no delay trials would have allowed us to examine the animal's decision time independent of the visual cue. Reaching movement to one of the two spatial targets could be made within 3 s. The touch on the chosen target was maintained for an initial holding-target period of 0.8 or 1.2 s and an additional visual feedback period of 0.8 or 1.2 s. Four feedback stimuli were used, which differed in shape or color. Two feedback stimuli (a 19.3 ° × 15.9 ° blue triangle and a 16.7° empty white circle) signaled the incorrect choice and two others (a 19.3 ° × 15.9 ° red triangle and an empty 14.8 ° × 14.3 ° white rectangle) signaled the correct choice. In each block of 21 correct trials, the correctness and incorrectness feedback stimuli were presented in pairs: the blue and red triangles were always presented in the same block, and the white circle and rectangle were presented in other blocks (Figure 1A, right panel). Next, a liquid reward (water) was delivered in correct trials. The volume per trial was the same for each session and every day. In error trials, the reward was not delivered. Each trial was followed by a period of 1-1.5 s intertrial interval, during which the video screen was black. In each trial, after a successful trial, the previously chosen target would reappear on the screen in the same position along with another target that could appear in one of three other possible positions, selected pseudo-randomly. The new target position could also be the same one not chosen in the previous trial. For the trial to be considered correct, the new target had to be chosen. If the target chosen in the previous trial was chosen again in the current trial, the trial was considered incorrect, and the reward was not delivered. After an error trial, a correction trial followed, in which the same two positional targets presented in the immediately preceding trial performed incorrectly were re-presented. An error in a correction trial was followed by another correction trial. The first choice of every session was always accepted as correct, and the reward was delivered for any chosen target.

#### Interaction with human agents

During the recording sessions (monkey P = 23; monkey C = 32) monkeys interacted with human partners. In each session, each monkey could potentially interact with three different human agents, who took turns one at a time. Two human agents, the so-called ‘Good Agents’ performed always correct trials, and one, the so-called ‘Bad Agent’ performed always incorrect trials.

The number of trials performed by the human agent was determined by the good human agent itself and was not predetermined. Both monkeys allowed the good human agent to perform multiple trials in a row. We were then able to increase the number of trials performed by the human agent and reduce the probability that the monkey could predict when the human's last action would be, thus keeping its level of attention and monitoring higher. The same was not true for the bad agent, as both monkeys were less tolerant of the human performing more than one trial in a row.

The alternation between human agents was random. The reward was always delivered to the monkey after a correct trial performed by the Good Agent. All three human agents did not always alternate in the same session. In some sessions, the monkey interacted with only two agents, either one good and one bad or with the two good ones. In other cases, only one agent interacted with the monkeys, either the good or the Bad Agent. In all cases, the human partner was sitting on the monkey’s right side, close to the animal. The human partner could start to perform the task only after the monkey completed a trial, indicating his turn by moving his hand toward the center of the screen only during the intertrial period when the screen was black, ready to touch the central stimulus when it appeared.

There were no other external cues that signaled the turn between the monkey and the human agent. After the human partner moved his hand toward the screen, monkeys were trained to let the human partner perform the trial without interfering. The Good Agent could perform a sequence from 1 to 4 trials, while the Bad Agent performed only one trial, each time. While the human performed the trial the monkey never interfered, and vice versa. Figure 1B shows an example of consecutive trials performed by both the monkey and the Good Agent (middle panel) or the Bad Agent (right panel). In all cases, when the human partner withdrew the arm at the end of a trial sequence, that was the signal for the monkeys to approach the central stimulus to begin a new trial. The interaction with the human started only after the monkeys had learned the task alone. The presence of a human as a partner reduced the likelihood of having predictive behavior in the observer monkey based on uncontrolled and uncorrectable stereotyped behaviors or eye or arm motor signals anticipated by another monkey as a partner. The human’s position at the monkey’s side, rather than in front, also prevented access to the human’s eye movements. During the task, the monkeys’ eye position was recorded but not placed under experimental control.

### Method details

#### Data collection

We used a non-commercial software package, CORTEX (http://www.nimh.nih.gov/labs-at-nimh/research-areas/clinics-and-labs/ln/shn/software-projects.shtml) to control stimulus presentation and reward delivery and to record touches on the screen and categorize the trials as correct or error, and based who was performing them, whether the monkey or the human. The eye position was monitored through the ViewPoint Eye Tracker system (Arrington Research, Scottsdale, USA) and recorded in the TDT system (Tucker-Davis Technologies, TDT, Alachua, USA). Extracellular neural activity was detected with a five-channel multi-electrode system (Thomas Recording, Giessen, Germany). Electrodes were quartz-insulated platinum-tungsten fibers (80-μm diameter, 0.8- to 2.5-MΩ impedance) and were inserted transdurally with microdrives (Thomas Recording). The raw signal was digitized at 24.4 kHz, and electrical signals were amplified and filtered. The LFPs were extracted from the raw signal (see Data analysis). For both time and frequency domain LFP analyses, linear trends were removed. Data analyses were performed using MATLAB (MathWorks, R2020a).

#### Data analysis

##### Behavior

We focused on two subsamples of sessions to compare 1) the human/monkey interactions between the two Good Agents and 2) the human/monkey interactions between Good Agents and Bad Agents. For the first comparison (Figure 2A), we analyzed only the recording sessions in which both Good Agent 1 (GA1) and Good Agent 2 (GA2) interacted with a monkey (monkey P = 7 sessions; monkey C = 13 sessions).

For the second comparison, only recording sessions in which both the Bad Agent (BA) and at least one Good Agent interacted with a monkey were analyzed (monkey P = 6 sessions; monkey C = 18 sessions; Figure 2B). In both analyses, we analyzed the percentage of correct trials and the response time (that is, the period that included the reaction and movement time) for humans and monkeys. We analyzed trials with a 1-second delay between the target presentation and the presentation of the go signal. For the Actor analysis, we included all trials in which the monkey performed the task himself. For the Observer analysis, we included all trials in which the human agent performed the task.

##### Neurophysiology

The dataset included 116 channels that were artifact-and noise-free in the voltage domain (monkey P = 50; monkey C = 66). These channels were further selected based on their LFP voltage visual response to the targets: i.e., we checked for signals that differed in voltage in the 300 ms time window, starting from 50 ms to 350 ms after the targets were presented, compared with the voltage in the baseline period of 300 ms just before the targets onset. Each channel was labeled as visual selective if it showed significant modulation in the 300 ms window either when the monkey was the Actor or when it was the Observer or both. Overall, we found 115 (monkey P = 49/50; monkey C = 66/66) visual selective channels out of 116 channels.

##### LFP spectrograms

Time-frequency analysis was computed using the multi-taper algorithm with the freeware toolbox “Chronux” (Chronux, version 2.00). For each trial, the mean value and linear trend were removed from the raw signal, and spectrograms were generated in a 300 ms window with 10 ms steps, using a frequency bandwidth of 5 Hz and 2 Slepian tapers. We set the maximum frequency to 100 Hz and obtained a time-by-frequency array, with frequency and time steps of 1.5 Hz and 10 ms, respectively. Relative spectrograms were defined as the ratio (in dB) between the power spectrum in each time-frequency bin and the mean power spectrum across all trials of baseline activity during the 300 ms prior to the targets' presentation. Each relative spectrogram corresponded to the average across all trials under the same conditions. Negative values of the normalized activities indicate lower activity compared to the baseline.

The analyses for the Good Agent and Bad Agent (Figures 5A and 5B) were performed by merging all the trials belonging to their type of interaction condition.

The GA1 and GA2 trials were merged to form a unique Good Agent type of interaction condition. For the Good Agents spectral comparison (Figure 4) and the Good Agents vs Bad Agent comparison (Figures 6A and 6B) we used the signal from the same subsamples of sessions used for the behavioral analyses. To avoid the bias introduced by sessions with too few and therefore unrepresentative trials, we only included sessions with at least 50 correct trials in each partner interaction (Average number of trials per interaction: Monkey 1, GA1 = 83.5 ± 17.2, GA2 = 91.9 ± 23.4, BA = 91.6 ± 14.2. Monkey 2, GA1 = 103.7 ± 22.7, GA2 = 95.0 ± 27.9, and BA = 107 ± 16.6).

The LFP bands were calculated by measuring the mean relative power belonging to the frequency range of the alpha (7-14 Hz), beta (20 – 30 Hz) and gamma band (45 – 100 Hz) from the corresponding relative spectrograms. For each time-frequency bin, comparisons between the LFP spectrograms were computed by measuring the mean relative power spectrum across trials and analyzing the difference between mean spectra for the conditions of each trial pair. We examined whether the differences were significant with separate permutation tests for each bin. The permutation test was performed by shuffling the power values across the two groups of trials, computing the difference in means between the reshuffled groups and repeating the shuffling process N times (N = 5000). For each comparison, we obtained a color-coded p-value map (p = 0.01) of differences by repeating the permutation test for all bins of the arrays. The overall significance was corrected by multiple comparisons using the false-discovery rate (FDR) method (Benjamini & Yekutieli, 2001; Durka et al., 2004), with an α- value of 0.01. Briefly, this “step-up” method was performed by: (1) ordering the p-values in an ascending series – p(1) ≤ p(2) ≤ … ≤ p(k); (2) finding the largest k for which p(k) ≤ αk/m; and (3) rejecting the null hypothesis for all bins with p ≤ p(k). The white color of the resulting time-frequency maps indicates p > 0.01 corrected by FDR; other colors indicate sign and intensity of significant differences.

##### Decoding procedure

To evaluate the classification accuracy with different randomly selected trials, we performed 1000 resampling runs. Each run generated a matrix with dimensions of n units by 32 trials (i.e., 8 trials for each correct target location in trial n-1). For decoding, we used a leave-one-trial-out approach. In this approach, one trial is designated as the test set, and all other trials form the training set. To maintain balance in the training set, three trials with different spatial coordinates from the tested target location trial were randomly selected and removed from the training set. For each time step, a regularized optimal linear estimator (RegOLE)^77^^,^^78^ associated the neural responses, consisting of a vector containing the Beta signal collected at each recording contact (15 for monkey P, 39 for monkey C), from a structure containing the signal of 28 correct trials (i.e., 7 trials for each target location) to the (x,y) coordinates of the associated target position for each of these 28 training trials. To avoid overfitting, we used a Tikhonov regularization, which yields the following minimization equation: norm(W∗(R+b)-C) + λ∗norm(W)). The scaling factor λ was chosen to provide a good trade-off between learning and generalization.^28^ Specifically, the decoder was constructed using two independent regularized linear regressions, one classifying the x-axis (two possible classes: -1 or 1) and one classifying the y-axis (two possible classes: -1 or 1). During testing, the output of the classifier was estimated for an n-element vector corresponding to the average neural activity on each recording channel for the time interval of interest on a test trial, new to the classifier. Classification accuracy was the average of the obtained proportion of correctly classified trials within each resampling run. To statistically evaluate the significance of the decoding accuracies, we used a one-tail nonparametric random permutation approach to estimate the 99.9% confidence interval limit. For each trial, we randomly reassigned its behavioral classification and recalculated the decoding accuracy. This process was iterated 1000 times, generating a distribution of accuracy values representing random performance. The accuracy of the real unpermuted data was considered significantly above chance if it fell within the 0.1% upper tail of its spatially defined random permutation distribution.
