# Supplementary material for: Simultaneous oscillatory encoding of “hot” and “cold” information during social interactions in the monkey medial prefrontal cortex
Source: iScience. 2024 Mar 25;27(5):109559. doi: 10.1016/j.isci.2024.109559 (PMC11033171; doi:10.1016/j.isci.2024.109559)
Supplement: Document S1. Figures S1–S5 and Data S1–S3 [file mmc1.pdf]

iScience, Volume *27*

Supplemental information

Simultaneous oscillatory encoding of “hot” and “cold” information during social interactions in the monkey medial prefrontal cortex

Fabio Di Bello, Rossella Falcone, and Aldo Genovesio

Data S1: Interactions length in human partners, related to Figure 1

Both Good Agents performed 1, 2, or 3 consecutive trials, and rarely 4 (GA1 34,0%, 39,0%, 26,9% and 0.1% with Monkey P and 32.2%, 43.0%, 24.6% and 0.2% with Monkey C; GA2 39,7%, 42,1%, 18,0% and 0,02% with Monkey P and 42.4%, 41.2%, 15.1% and 1.3% with Monkey C). The Bad Agent always performed 1 trial with both Monkey P and Monkey C. During the interaction with the Good Agents the monkeys mostly performed 1 (rarely), 2, 3, 4,5 or 6 consecutive trials (GA1 0.08%, 25.6%, 50.8%, 19.9%, 1.4% and 2.4% with Monkey P and 0.2%, 26.8%, 56.0%, 13.1%, 2.7% and 1.2% with Monkey C; GA2 2.7%, 25.8%, 38.7%, 19.4%, 8,9% and 4.6% with Monkey P and 1.5%, 33.1%, 40.5%, 15.7%, 5.0% and 4.3 with Monkey C). During the interaction with the Bad Agent, the monkeys usually performed 1 (rarely), 2, 3, 4, 5 or 6 consecutive trials (BA1 14.4%, 26.7%, 28.5%, 16.2%, 8.7% and 5.6% with Monkey P and 0.5%, 12.8%, 30.4%, 30.5%,15.6% and 10.2% with Monkey C).

Data S2: Monkey performance in Good and Bad interactions, related to Figure 2

The percentage of accuracy was well above chance in both the Bad Agent interaction (mean ± std, Monkey P, Acc Bad =93.0 ± 7.5; Monkey C, Acc Bad = 83.9 ± 14.9) and the Good Agent interaction (mean ± std, Monkey P, Acc Good1 = 91.8 ± 10.9, Acc Good2 = 80.1 ± 12.7; Monkey C, Acc Good1 = 85.3 ± 6.8, Acc Good2 = 85.5 ± 9.9) and did not differ from each other (Monkey P: Kruskal-Wallis nonparametric test, $p = 0.20$; Monkey C: Kruskal-Wallis nonparametric test, $p = 0.91$).

Data S3: Beta and Gamma results in Good and Bad interactions, related to Figure 4

Good Agents' interactions

We first investigated the beta and gamma dynamics when the monkey was the Actor or the Observer in the interaction with the Good Agent. All sessions in which the monkey worked with at least one Good Agent were included in this analysis.

<u>Monkey as Actor</u>

Overall, both beta and gamma showed an opposite trend of their relative power modulation, thus resulting in a strong anti-correlation between the two signals in both monkeys (monkey P: $r = -0.94$, $p < 0.001$; monkey C: $r = -0.45$, $p < 0.001$).

After the presentation of the target, the gamma relative power rapidly increased in both monkeys (Fig. 4A, red arrows in the left panels; monkey P: 1st peak ~230 ms; monkey C: 1st peak ~110 ms), followed by a tonic decrease. A second, small increase of gamma activity was found immediately after the reaching in monkey P (Fig. S3A, red arrow in the left panel; 2nd peak ~10 ms, Mov Onset aligned), and before the movement onset in monkey C (Fig. S3A, red arrow in the left panel; 2nd peak ~ -120 ms, Mov Onset aligned). Comparing the gamma peaks after target presentation with those emerging around movement onset, the former was higher in both monkeys (monkey P: Kruskal–Wallis nonparametric test, $p < 0.001$; monkey C: Kruskal–Wallis nonparametric test, $p < 0.001$). Later in the trial, the gamma (or beta) band almost reached the activation levels as seen in the pre-target onset period (Fig. 4A).

As opposed to gamma, the beta band rapidly decreased in response to the targets by peaking negatively at ~280 ms in monkey P and ~270 ms in monkey C (Fig. 4A, blue arrows in the left panels). After, the beta relative power showed a sharp positive rebound that lasted until ~750 ms, followed by a second deflection that peaked negatively before the motor response in monkey P (Fig. S3A, blue arrow in the left panel; 2nd peak ~ -250 ms, Mov Onset aligned) and immediately after the motor response in monkey C (Fig. S3A, blue arrow in the left panel; 2nd peak ~20 ms, Mov Onset aligned). In both monkeys, the first beta deflection in response to the target presentation was more pronounced than the subsequent one (monkey P: Kruskal–Wallis nonparametric test, $p < 0.001$; monkey C: Kruskal–Wallis nonparametric test, $p < 0.001$). Later in the trial, the beta band increased again by showing a higher relative power than the gamma band. In monkey P such beta overtaking occurred before the movement time, while in monkey C it appeared later.

<u>Monkey as Observer</u>

When the monkey was the Observer, both beta and gamma bands showed opposite power modulations, thus resulting in a strong anti-correlation between the two signals in both monkeys (monkey P: $r = -0.54$, $p < 0.001$; monkey C: $r = -0.34$, $p < 0.001$).

The gamma relative power rapidly increased in both monkeys in response to the target presentation (Fig. 4A, red arrows in the right panels; monkey P: 1st peak ~250 ms; monkey C: 1st peak ~100 ms), followed by a tonic decrease. After the go onset, the signal increased again by peaking after the movement and before the target touching of the human partner (Fig. S3A, red arrows in the right panels; monkey P: 1st peak ~220 ms; monkey C: 1st peak ~20 ms, Mov Onset aligned). This second peak was significantly higher than the first in both monkeys (monkey P: Kruskal–Wallis nonparametric test,

p < 0.05; monkey C: Kruskal–Wallis nonparametric test, p < 0.01). Later in the trial, the gamma (or beta) band almost reached the activation levels as seen in the pre-target onset period (Fig. 4A).
The beta band decreased in response to the targets by peaking negatively at ~300 ms in monkey P and ~270 ms in monkey C (Fig. 4A, blue arrows in the right panels). Following this, the beta relative power displayed a positive rebound that persisted until the go-signal presentation. This was followed by a negative peak occurring after the movement onset but before the human partner touched the target (Figure S3A, blue arrows in the right panels; monkey P: 2nd peak ~90 ms; monkey C: 2nd peak ~170 ms, Mov Onset aligned). There was no significant difference between the two consecutive Beta deflections in both monkeys (monkey P: Kruskal-Wallis nonparametric test, p = 0.14; monkey C: Kruskal-Wallis nonparametric test, p = 0.61). Later in the trial, the beta band activity increased again in monkey P, while it returned to baseline levels in monkey C.
Thus, in the actor role there was a strongest opposite beta/gamma response to the target presentation, whereas in the observer role the strongest opposite beta/gamma response appeared after the onset of movement but before the human partner touched the target. In both monkeys, the event-locked fluctuation emerging around the reaching movement was delayed in the observer role compared to the actor role.

Bad Agents' interactions

We analyzed the LFP neural dynamics characterizing the interaction with the Bad Agent, both when the monkey was the Actor and the Observer. All sessions in which the monkey worked with the Bad Agent were included in this analysis.
<u>Monkey as Actor</u>
In general, the beta and gamma bands showed modulations of their relative power in opposite directions, thus resulting in a strong anti-correlation between the two signals in both monkeys (monkey P: r = -0.86, p < 0.001; monkey C: r = -0.46, p < 0.001).
After the target presentation the gamma band rapidly increased in both monkeys (Fig. 4B, red arrows in the left panels; monkey P: 1st peak ~290 ms; monkey C: 1st peak ~110 ms). Thereafter, a tonic decrease began and it was interrupted by an increase in activity that emerged between the go-signal and the movement onset (Fig. S3B, red arrows in the left panels; monkey P: 2nd peak ~ -230 ms; monkey C: 2nd peak ~ -80 ms, Mov Onset aligned). In both monkeys, the gamma peaks that appeared after target presentation were higher than those that appeared around the beginning of movement (monkey P: Kruskal–Wallis nonparametric test, p < 0.01; monkey C: Kruskal–Wallis nonparametric test, p < 0.001). Later in the trial, the gamma (or beta) band almost reached the activation levels as seen in the pre-target onset period (Fig. 4B).
The beta band rapidly decreased in response to the targets by peaking negatively at ~250 ms in monkey P and ~280 ms in monkey C (Fig. 4B, blue arrows in the left panels). After, the beta relative power showed a sharp positive rebound followed by a second deflection that peaked negatively before the movement onset in monkey P (Fig. S3B, blue arrow in the left panel; monkey P: 2nd peak ~ -100 ms, Mov Onset aligned) and immediately after the onset of the movement in monkey C (Fig. S3B, blue arrow in the left panel; monkey C: 2nd ~ 80 ms, Mov Onset aligned). Both monkeys showed a more pronounced first beta deflection in response to the target presentation compared to the second deflection that emerged around movement onset (monkey P: Kruskal-Wallis nonparametric test, p < 0.001; monkey C: Kruskal-Wallis nonparametric test, p < 0.001). Later in the trial, the beta band showed a higher relative power than the gamma band.
<u>Monkey as Observer</u>
The beta and gamma bands were strongly anti-correlated in both monkeys (monkey P: r = -0.41, p < 0.001; monkey C: r = -0.21, p < 0.002).
Initially, the gamma relative power showed an increase in response to the target onset in both monkeys (Fig. 4B, red arrows in the right panels; monkey P: 1st peak ~550 ms; monkey C: 1st peak ~40 ms). A second peak of gamma also emerged after the motor response (Fig. S3B, red arrows in the right panels; monkey P: 2nd peak ~320 ms; monkey C: 2nd peak ~230 ms, Mov Onset aligned). This second peak was significantly higher than the first one in both monkeys (monkey P: Kruskal–Wallis nonparametric test, p < 0.05; monkey C: Kruskal–Wallis nonparametric test, p < 0.001).
Opposite to the gamma activity, the beta band showed two consecutives negative peaks in response to the targets' presentation (Fig. 4B, blue arrows in the right panels; monkey P: 1st peak: ~290 ms; monkey C: 1st peak: ~310 ms) and following the starting of the movement of the human partner (Fig. S3B, blue arrows in the right panels; monkey P: 2nd peak: ~270 ms; monkey C: 2nd peak: ~290 ms, Mov Onset aligned). In both monkeys, the two successive Beta negative peaks did not show a significant difference (monkey P: Kruskal-Wallis nonparametric test, p = 0.95; monkey C: Kruskal-Wallis nonparametric test, p = 0.61). Later, the beta band reached the baseline level.
Thus, in the actor there was a strongest opposite beta/gamma response to the target presentation, while in the observer a strongest opposite beta/gamma response after the onset of movement but before the human partner touched the

target. In both monkeys, the event-locked fluctuation emerging around the reaching movement was delayed in the observer role compared to the actor role.
Overall, we observed distinct modulations of beta and gamma neural networks associated with social roles. In the Actor role, there was increased activation of both beta and gamma resources in the early phase of the trial, whereas the Observer role showed greater gamma engagement in the later part of the trial compared to the early phase.
When comparing the latencies around movement onset between the Actor and Observer in both interaction types, although the order of neural response offsets differs between the two monkeys (i.e., the beta peak precedes the gamma peak in Monkey P, while the opposite is observed in Monkey C), we found that both beta gamma responses peaked earlier in the Actor role compared to the Observer role in both monkeys (Kruskal-Wallis nonparametric test, $p < 0.001$ for both monkeys in both interaction types). Overall, the event-locked fluctuations that occurred around the reaching movement were delayed in the observer role compared to the actor role.
The presence of common response patterns (within Actor and Observer) strongly suggests that the two frequency bands represent equivalent functional networks in both monkeys, despite the differences in the signals. Indeed, frequency differences between different animals expressing the same functional neural network are not uncommon in the literature [1–3].

References

1. Buzsáki, G., Logothetis, N., and Singer, W. (2013). Scaling Brain Size, Keeping Timing: Evolutionary Preservation of Brain Rhythms. Neuron *80*, 751–764. 10.1016/j.neuron.2013.10.002.

2. Reinhart, R.M.G., Heitz, R.P., Purcell, B.A., Weigand, P.K., Schall, J.D., and Woodman, G.F. (2012). Homologous Mechanisms of Visuospatial Working Memory Maintenance in Macaque and Human: Properties and Sources. J. Neurosci. *32*, 7711–7722. 10.1523/JNEUROSCI.0215-12.2012.

3. Stoll, F.M., Wilson, C.R.E., Faraut, M.C.M., Vezoli, J., Knoblauch, K., and Procyk, E. (2016). The Effects of Cognitive Control and Time on Frontal Beta Oscillations. Cereb. Cortex *26*, 1715–1732. 10.1093/cercor/bhv006.

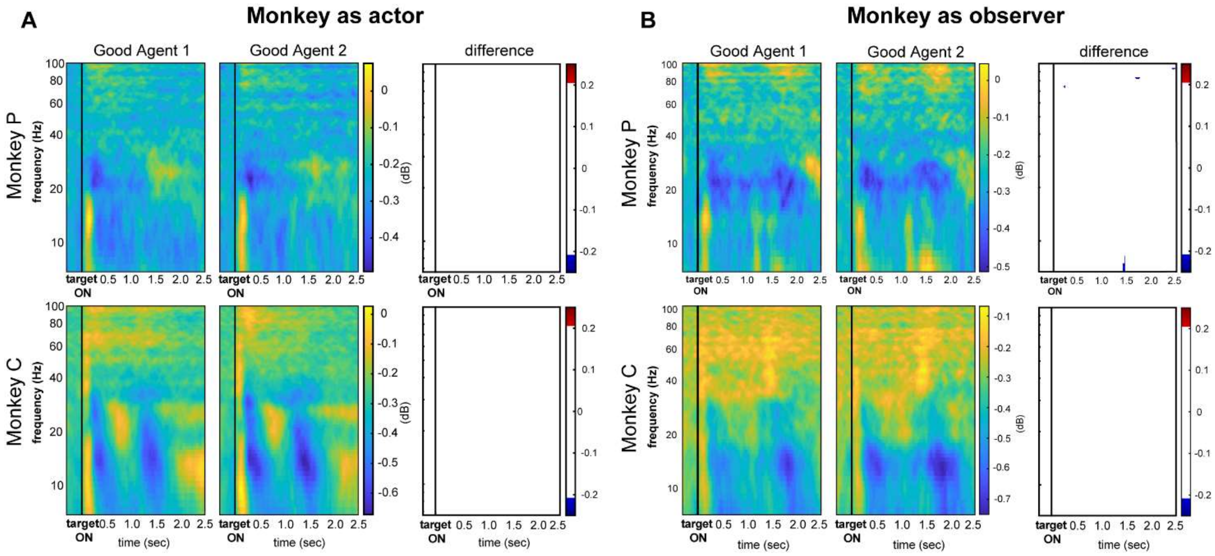

Figure S1. Grand average time-frequency plot of Actor and Observer in interaction with Good Agents. Related to Figure 4, Figure 5 and Figure 6. The average relative spectrograms, normalized to baseline, of the raw LFPs of both monkey P (top panels) and monkey C (bottom panels) during the interaction with GA1 (left spectrograms) and GA2 (right spectrograms) are shown when the monkey is the Actor (A) and the Observer (B). The rightmost maps in A-B show the difference between the spectrograms (GA1 minus GA2). Colored pixels represent statistically positive differences (red color scale; $p < 0.01$) or negative (blue color scale; $p < 0.01$).

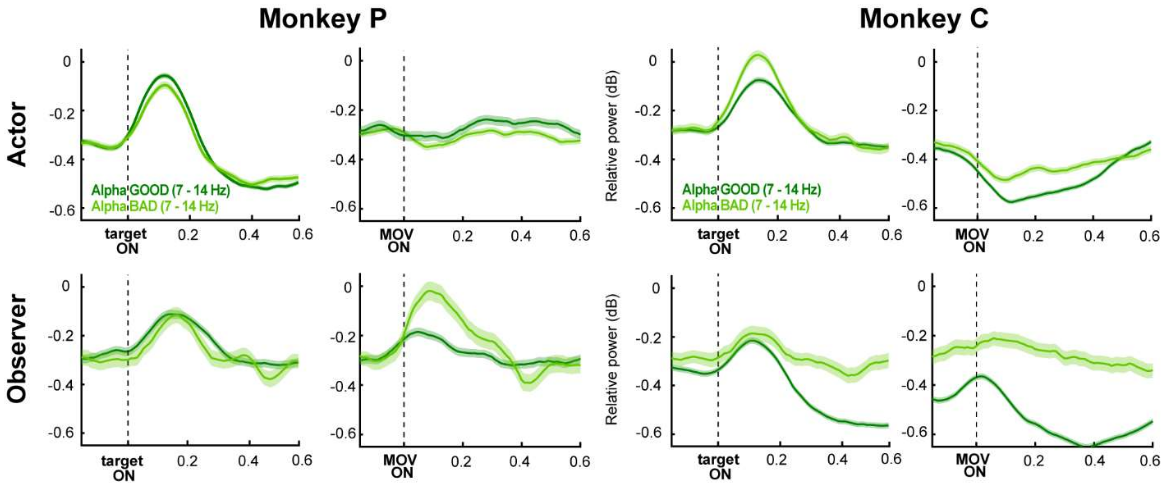

Figure S2. Alpha-band dynamics of social roles during interaction with Good and Bad Agent. Related to Figure 4. Average relative spectrograms, normalized to baseline, of the alpha band when the monkey was the Actor and the Observer during the interaction with the Good Agent (green) and the Bad Agent (light green). The vertical dashed lines correspond to the average Movement onset (Mov).

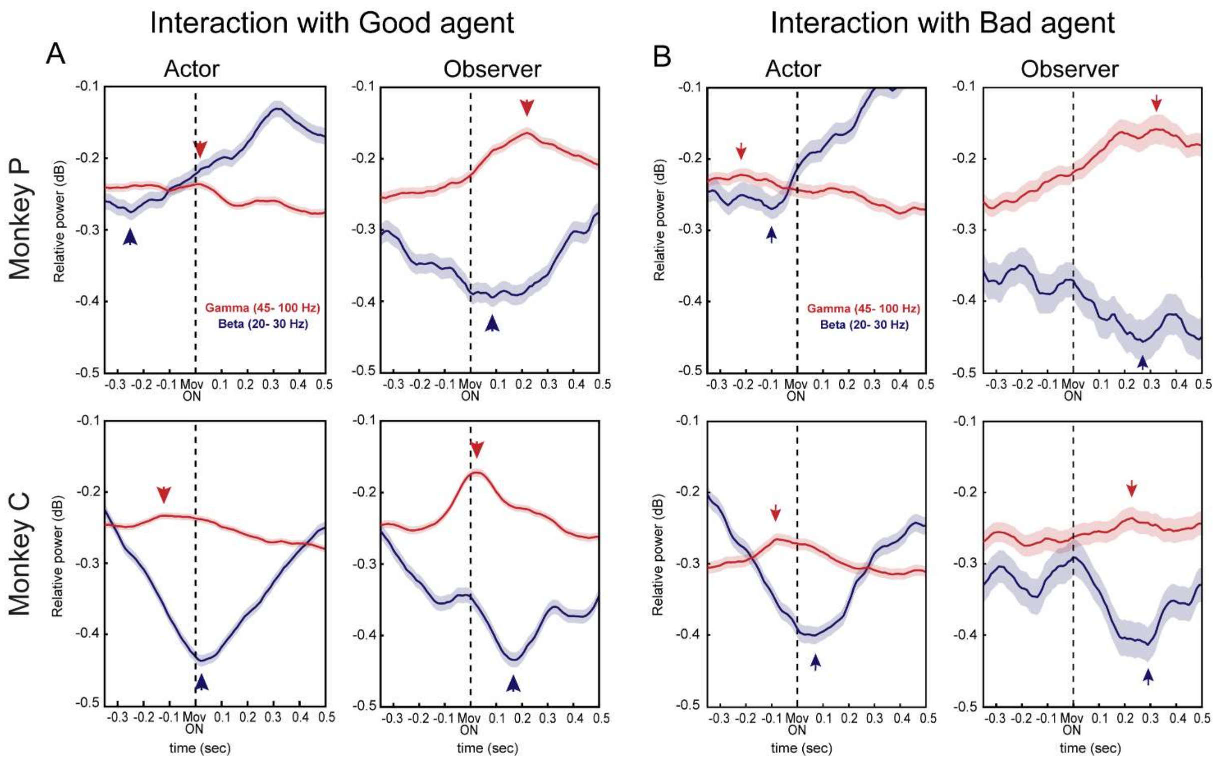

Figure S3. Beta and gamma bands dynamics of social roles in different types of interaction. Related to Figure 4. Average relative spectrograms, normalized to baseline, of beta (blue) and gamma (red) bands when the monkey is the Actor and the Observer during the interaction with the Good Agent (A) and the Bad agent (B), respectively. The vertical dashed line indicates the movement onset time (time 0). The vertical blue (red) arrows represent the local minimum (maximum) of beta (gamma) across the time window displayed.

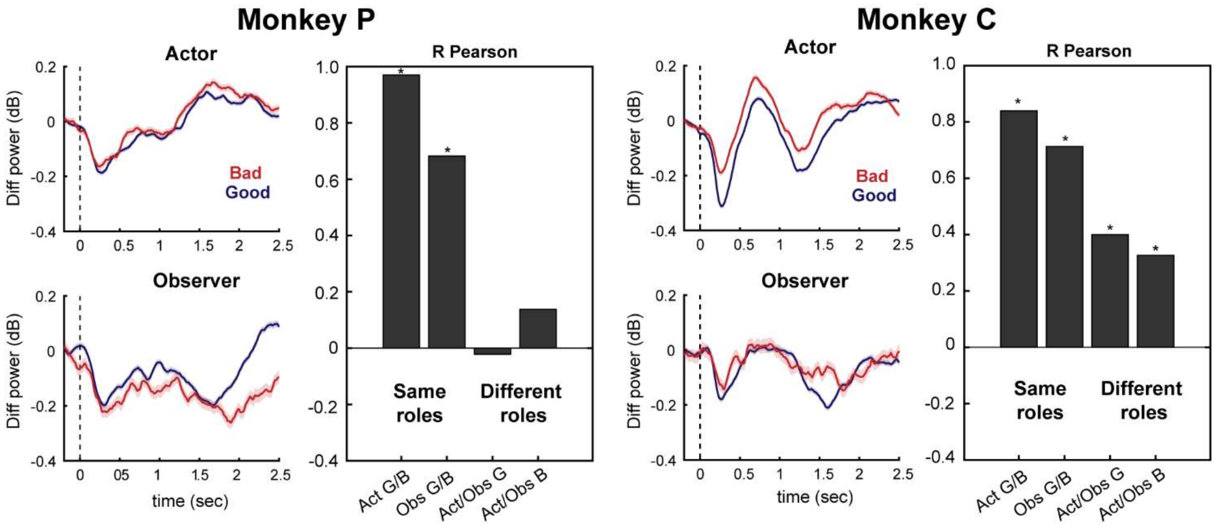

Figure S4. Related to Figure 4. For both Monkey P and Monkey C, the left panels show the average relative spectrogram differences between the beta and gamma bands during interactions with the Good Agent (blue) and the Bad Agent (red) when the monkey is in the role of Actor (top panel) and Observer (bottom panel). The right panels show the Pearson's correlation coefficient values of the beta/gamma differences for the same roles in different interactions (Actor Good vs. Actor Bad and Observer Good vs. Observer Bad) and for different roles in the same interaction (Actor Good vs Observer Good and Actor Bad vs Observer Bad). Asterisks denote significant correlations.

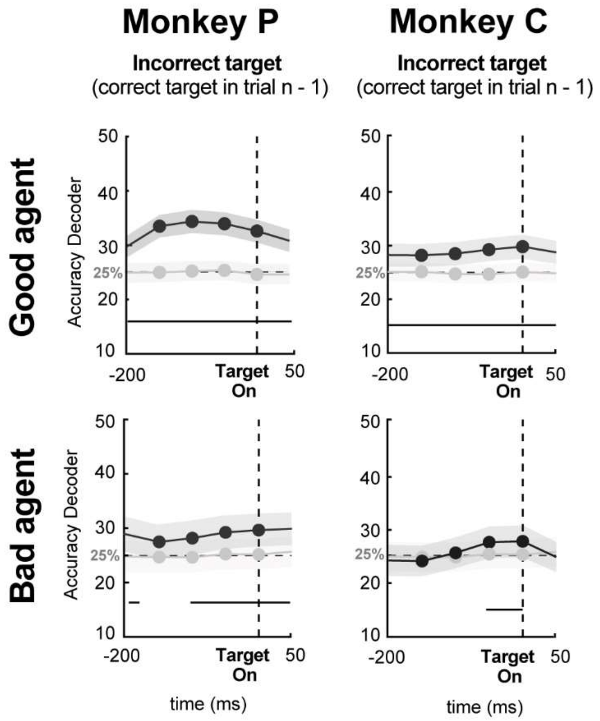

Figure S5. Related to Figure 4. Decoding accuracies obtained from beta band activity. Lines and shading show mean ± SE classification accuracy around the target onset for the selection (dark gray) and randomized (light gray) distributions. The distribution reflects 1,000 iterations of classifiers trained and tested on n = 8 randomly sampled trials. Horizontal bars (top right of each plot) indicate the above chance classification (permutation test, $p < 0.01$). The chance level is at 25%, as the horizontal dashed line represents.
